# Supplementary material for: Cytotoxic Fractions from Hechtia glomerata Extracts and p-Coumaric Acid as MAPK Inhibitors
Source: Molecules. 2021 Feb 19;26(4):1096. doi: 10.3390/molecules26041096 (PMC7922350; doi:10.3390/molecules26041096)
Supplement: Supplementary file 1 [file molecules-26-01096-s001.pdf]

Table S1. Up-regulated genes (Zscore >2).

Cy3	Cy5	Id	Symbol	Zscore
203	1050	H300021260	PKHD1	4.197642876
527	3002	OPHSV0400006985	-	4.06089168
396	2850	OPHSV0400013248	-	4.000444642
222	748	H300001106	-	3.979742155
248	829	H200000432	CYP4A11	3.957856997
2967	15967	OPHSV0400003849	NP_997263.1	3.657889374
7559	40397	OPHSV0400007038	5S_rRNA	3.571565371
235	811	OPHSV0400001730	CALN1	3.518902934
1818	7064	OPHSV0400001008	NP_060219.2	3.457328872
318	1464	H200008732	RMI1	3.420308897
228	561	OPHSV0400012700	-	3.415107619
196	707	OPHSV0400007004	-	3.376651926
253	747	H200018901	-	3.365692538
211	474	H300002231	PHLDA1	3.169186439
2612	8323	H300015725	BRPF3	3.119353024
407	1040	OPHSV0400003148	FAM124B	3.119203754
367	890	H300009173	CITED4	3.118073733
586	1464	H300007352	Q9P135_HUMAN	3.078619805
398	1265	OPHSV0400003294	NP_997198.2	3.04104777
295	739	H300019922	THRA	3.027837267
196	409	H200000200	EBI2	3.016724915
290	765	OPHSV0400005297	HIST1H4A	2.985334216
241	694	H200002018	CCDC113	2.98319359
243	651	H300007721	NEUROG2	2.95827258
253	567	OPHSV0400002253	BGN	2.920762416
219	496	OPHSV0400001221	DDX23	2.920417728
2137	6810	OPHSV0400000970	FAM20C	2.912919839
4573	15180	OPHSV0400006736	-	2.911519871
714	2487	H300000423	IKBKG	2.896676276
242	657	OPHSV0400008374	-	2.888697689
201	442	OPHSV0400006180	IFI16	2.884925627
215	484	H300003346	Q86V52_HUMAN	2.88490925
372	937	H300003104	SUSD2	2.882105684
1030	3203	H300007913	NP_689812.2	2.861710135
6219	18144	H200003429	ARRDC3	2.853707604
235	504	OPHSV0400000611	RYK	2.849211867
375	1078	H200016066	PRLHR	2.847187639
939	3089	H300003340	TIMM10	2.845571417
305	731	H300022808	ATP9B	2.83817713
363	969	OPHSV0400008294	-	2.83417112
606	1331	ALIEN6_90	-	2.830520165
211	454	OPHSV0400006867	-	2.81785417
395	848	OPHSV0400000614	ZNF589	2.810588405
258	597	H300021048	API5	2.802783271
1637	5605	OPHSV0400007952	-	2.79987006
442	950	OPHSV0400002863	-	2.798130839
222	543	OPHSV0400004393	Q8TCQ8_HUMAN	2.780992881
225	493	H200006531	SERPINI1	2.779826305
293	659	H200016513	MKNK2	2.761233814
521	1481	H200002849	C1orf35	2.740436636
867	2653	OPHSV0400006637	C6orf141	2.733036849
1244	3756	OPHSV0400011008	SNX12	2.723633198
236	525	OPHSV0400000879	ZC3H11A	2.714574066
194	452	H200000567	BLK	2.705789855
693	1895	OPHSV0400006363	GDE5_HUMAN	2.704412506
543	1452	H300006692	Q3SXP8_HUMAN	2.704142065
339	725	H300011400	C1orf9	2.703356562
719	1860	OPHSV0400008722	LCN8	2.696300919
1961	5025	OPHSV0400010160	-	2.687096704

235	523	OPHSV0400010183	-	2.687010946
214	474	H300014307	RHAG	2.68117663
284	563	H200008321	NP_079105.4	2.676979248
197	415	H200017053	ERAF	2.675473959
326	807	H300021927	MAK10	2.656682424
1410	3837	OPHSV0400009700	-	2.65055643
311	716	OPHSV0400013405	-	2.650347678
567	1267	H200017407	ANGPT4	2.649571389
1080	3347	H300006583	SLC9A10	2.649039708
200	369	OPHSV0400008485	-	2.641674637
260	569	H200014546	PLCH1	2.637987591
286	605	H200007482	SERPINA4	2.628269552
602	1751	H300001220	SLITRK6	2.616990317
236	448	OPHSV0400007772	-	2.6044361
206	479	OPHSV0400012363	-	2.593223532
216	376	OPHSV0400009721	-	2.590417979
387	1198	OPHSV0400009912	-	2.582757898
219	393	H300015718	ABHD6	2.580406879
399	818	H300001569	ATF6B_HUMAN	2.580158995
247	642	H300020108	ANKRD10	2.579947683
192	406	OPHSV0400002467	TBX1	2.579541711
285	564	OPHSV0400002812	-	2.574563409
32679	62125	H300009305	UBE2U	2.570985392
300	701	OPHSV0400011253	-	2.5683736
1632	4512	OPHSV0400006811	CACNA1G	2.564830532
234	478	OPHSV0400011244	-	2.555338457
688	1953	OPHSV0400000310	GABRA3	2.54548961
275	623	H300009838	IL1RAPL1	2.542478971
449	1274	H200002292	NP_056996.2	2.535421991
199	436	OPHSV0400003474	-	2.534976717
208	475	H200015822	ABCA11	2.534939501
727	1768	H300015139	CLSTN3	2.523457514
1193	3073	OPHSV0400000064	BNIP3L	2.522039971
4876	12820	H200021247	SPESP1	2.515393029
255	527	H200020812	TMEM83	2.510468188
1481	3456	H200008294	PM14_HUMAN	2.503070998
229	440	OPHSV0400011168	-	2.500349263
229	440	OPHSV0400009889	-	2.496946051
375	947	H200001239	MON1A	2.492038247
261	497	OPHSV0400010226	SRA1	2.48351024
263	566	OPHSV0400001707	GPR109A	2.481992967
262	642	OPHSV0400002581	Q5BKX7_HUMAN	2.479815195
314	631	OPHSV0400010156	RASA4	2.479794934
1220	3075	H200016227	KLHL26	2.479216541
299	601	OPHSV0400001801	SETD3	2.475277523
215	451	OPHSV0400010442	-	2.475116259
238	465	H200010981	ZNF750	2.474366702
615	1294	H300019935	WHSC1	2.467395786
313	542	OPHSV0400010838	-	2.466145765
217	375	H200002403	NAT8B	2.464960031
413	833	H200006342	FBXO8	2.460891743
740	1760	OPHSV0400009476	SAE2	2.458622301
266	544	OPHSV0400008978	NP_001013737.1	2.457483658
396	792	H300021170	ATXN2L	2.456335392
571	1572	H200008552	CC2D1A	2.449490312
270	565	OPHSV0400004153	EVC2	2.445456787
545	1304	OPHSV0400011315	-	2.438890908
318	618	H300016197	ICOS	2.438734782
207	361	H300003393	NP_710156.2	2.435207239
712	1901	OPHSV0400008730	-	2.435158443
220	410	H300008010	Q8NGD7_HUMAN	2.431043671
399	1002	OPHSV0400008581	-	2.430564904
602	1259	OPHSV0400001538	-	2.429298016
451	1022	H200000053	Q15109-2	2.429067604

384	883	H200009532	SLC35B4	2.428684827
252	523	OPHSV0400002303	NDN	2.425724386
347	740	OPHSV0400009523	-	2.424909077
1831	5556	H200009998	FARP2	2.421709512
220	410	H200000724	TNP1	2.419179896
1679	4279	H300021503	ARSJ	2.41865852
384	926	H200000101	SLC1A2	2.413122364
509	1205	OPHSV0400009427	-	2.40773825
257	516	H200004859	F10	2.403530559
243	436	H300019111	CHURC1	2.403180922
729	1655	OPHSV0400011242	Q71RF5_HUMAN	2.394863287
285	646	OPHSV0400000225	PPP1R3D	2.393744178
14013	32173	H300020243	SRP9	2.393663822
331	691	OPHSV0400013229	-	2.389423446
294	590	OPHSV0400009171	-	2.383938585
398	1024	H200003502	CENTD3	2.382302076
215	422	H200001478	TTC1	2.381392498
284	572	H300014391	TRIM6	2.381123727
457	1048	H200009547	TMEM182	2.37579893
602	1445	OPHSV0400006300	VPS36	2.373826946
715	1994	H300021145	ANGPTL4	2.372667897
691	1672	H300019488	TBN	2.3709945
229	374	H300008795	-	2.369933171
294	709	H200014979	LCN2	2.360788515
27058	56397	H300007858	-	2.358712496
269	582	OPHSV0400006708	SPTBN1	2.35703686
471	1223	OPHSV0400012246	-	2.35539745
1615	4351	H200021204	NLRP4	2.352206811
208	358	H200004931	PPP2R3C	2.350761682
2087	4801	OPHSV0400010176	-	2.345998975
3864	10751	OPHSV0400012406	-	2.344201278
203	427	OPHSV0400012941	-	2.341800024
217	411	H200003755	MED31	2.340843223
310	626	H300000796	ZNF497	2.33838446
2875	7785	H300010020	GSTA3	2.336416135
914	2296	H200013400	T	2.335110117
242	461	H200016529	HAL	2.321716244
301	544	OPHSV0400010235	-	2.320911518
401	803	H300018888	BNIP1	2.320113584
278	652	OPHSV0400012845	-	2.319513137
1199	3040	H300016440	TRIM9	2.319041567
385	761	OPHSV0400004074	DGKG	2.315105842
222	364	H300006941	-	2.31399991
261	501	OPHSV0400012851	-	2.313354522
218	406	H300002301	HES4	2.312194105
4164	11906	H300003567	Q96JN9_HUMAN	2.310302676
197	330	H200007362	CCDC19	2.303897607
223	382	H300017665	C22orf28	2.30157112
735	1783	OPHSV0400007118	-	2.297797887
257	513	OPHSV0400007096	ZNF321	2.296983157
701	1984	H200017287	MATN3	2.293650899
199	386	OPHSV0400012841	-	2.290916666
11805	30482	H300011097	SH2B1	2.289877734
216	433	OPHSV0400002912	-	2.288805819
274	547	OPHSV0400008420	-	2.287719266
8835	20862	H300003153	WIF1	2.283061712
1467	3642	H300018839	ISCU	2.28231848
1091	2530	OPHSV0400008575	-	2.279873005
307	581	H200014420	TMED8	2.277962038
420	789	OPHSV0400000673	-	2.276984034
427	964	H300020299	Q96CK5_HUMAN	2.276593246
2041	4223	H300011665	-	2.274549431
212	465	OPHSV0400006627	NUP62	2.271228261
2027	5522	OPHSV0400002916	NP_114110.1	2.271062295

2570	6248	OPHSV0400005502	-	2.270715552
242	498	H300019985	STK3	2.269148638
245	449	OPHSV0400013250	-	2.267625321
513	1048	OPHSV0400011170	-	2.263340893
362	815	H200010231	MED18	2.260548758
1490	3307	OPHSV0400003792	ZNF613	2.258306901
209	396	H200007883	GATA3	2.257392911
1979	4260	H300011700	GPHN	2.255929828
2529	6293	OPHSV0400004818	-	2.255630209
348	747	OPHSV0400001749	IGLV7-43	2.253996882
390	915	H200017696	PCDHA13	2.253542246
2025	5367	OPHSV0400000598	-	2.252658301
342	691	M2NC000005	-	2.250941187
248	453	H200001480	MRPL27	2.250642998
203	324	OPHSV0400000619	INTU	2.249757666
270	539	H200003905	EYA2	2.24886062
472	955	OPHSV0400013093	-	2.245575352
434	867	H300016327	-	2.244296464
1108	3022	H300015926	CLPB	2.241848256
725	1513	OPHSV0400003037	UBE2A	2.239652738
225	361	OPHSV0400012556	-	2.239494083
1187	3332	H200000856	PSMD1	2.239049035
1019	2487	H200013233	AP3B2	2.237209287
539	1375	OPHSV0400006699	-	2.23412734
412	897	H200017509	PJA2	2.231262346
736	1661	H300002735	OSR1	2.22965988
204	360	H300005750	HSPA5	2.229463984
371	753	OPHSV0400009917	-	2.22857674
421	865	OPHSV0400007766	TRIM61	2.227943173
442	813	OPHSV0400012166	CENPP	2.226095802
1184	2356	H300012564	HNRPDL	2.224145378
668	1516	H300018268	NP_694996.3	2.223299079
273	519	H200010976	-	2.217351185
2691	6286	OPHSV0400009221	-	2.216866173
251	425	H200003730	GMPPA	2.215233395
195	339	H300022389	ATXN1	2.213995647
236	427	OPHSV0400004447	IRF4	2.212767572
325	665	OPHSV0400001160	ZNF273	2.202411282
1499	3330	H300014804	SEC31A	2.201843106
244	567	OPHSV0400003788	-	2.201393786
201	323	OPHSV0400008640	NP_001019848.1	2.199564899
195	350	OPHSV0400001235	DEFB112	2.199541179
307	585	H300018420	ERCC1	2.199434103
270	559	H300021717	NAGPA	2.198057239
369	661	H200004545	ZNF45	2.196673284
1388	3280	H200013791	KIAA0100	2.196063521
248	469	H300013173	AXUD1	2.195933145
268	558	H300019838	SELP	2.1957967
422	930	OPHSV0400001885	-	2.194062516
932	1830	H200019953	MTHFSD	2.192931593
484	1117	OPHSV0400004103	ALG1	2.191871242
890	1647	H200019571	ZNF20	2.191425989
297	597	OPHSV0400005037	ZNF524	2.190351386
2103	5618	OPHSV0400007617	IKZF1	2.188966179
256	486	OPHSV0400010490	-	2.187824517
207	405	OPHSV0400010645	-	2.186678466
382	780	OPHSV0400001030	AQP6	2.18388317
3870	10378	H300001674	FEZF1	2.183684339
277	489	H200007026	GNG11	2.183567754
341	677	H200005557	RNF113A	2.183461777
1133	2343	OPHSV0400002058	NP_001030177.1	2.183453661
404	920	OPHSV0400011345	-	2.181921913
245	511	OPHSV0400004966	C14orf83	2.177847498
685	1442	OPHSV0400007156	-	2.176899093

243	497	OPHSV0400004155	-	2.176645531
872	2154	H200004752	PPP1R16B	2.175426735
899	2119	OPHSV0400003874	OX26_HUMAN	2.175099968
575	1279	OPHSV0400005909	ALDH3B2	2.173527215
4690	12323	H300018592	ANGPT2	2.173439739
2380	5086	OPHSV0400012961	NEO1	2.172374201
484	986	H300020748	SH3GLB2	2.171220574
932	2336	H300016947	MYCN	2.170954651
497	1031	OPHSV0400008729	NP_001005751.1	2.169226344
319	708	OPHSV0400000988	-	2.168639501
268	566	H200003419	C2orf44	2.168578499
1848	4120	H200011229	C14orf43	2.168144577
679	1497	H200014421	SALL4	2.166163714
219	362	OPHSV0400008150	-	2.165329846
6322	14576	H200005173	ZNF165	2.16485818
717	1612	OPHSV0400010150	C5orf13	2.164491844
2152	5234	H300002425	CNTNAP2	2.161772348
586	1398	OPHSV0400004877	MEF2C	2.160931841
1492	3849	H300006097	CCL17	2.159641754
729	1639	OPHSV0400002624	UBE1L	2.159611763
406	840	OPHSV0400009342	CPSF1	2.156061985
244	466	OPHSV0400001315	ASCC3	2.155749562
3660	8559	H300007751	FGL1	2.151986326
296	595	H200012177	RAB4A	2.15115472
522	1114	OPHSV0400003783	-	2.148802276
658	1206	H300009858	SHPRH	2.146632206
446	857	H300021455	STARD10	2.146207812
949	1819	OPHSV0400009177	-	2.145067462
2418	5833	OPHSV0400011623	-	2.144919365
2060	5447	H300020383	ZNF7	2.144625346
245	473	OPHSV0400007668	-	2.143173142
434	790	H300022109	GAPVD1	2.143073891
2816	5887	H300008847	C1QL3	2.142755831
381	703	H200002321	ART4	2.140384047
307	584	OPHSV0400002500	C10orf72	2.139863541
319	548	H200001917	CSRP2	2.138943976
523	1025	H200008828	C10orf12	2.133880051
874	1877	H300022748	CADPS	2.133082592
869	1683	OPHSV0400004329	KCNMA1	2.13136001
4239	9658	H200005036	TRIM58	2.131036738
193	363	H200010853	C1orf158	2.130727786
3338	7454	OPHSV0400001898	C14orf80	2.130023033
248	505	H200003117	-	2.130016665
468	946	H200001477	C14orf11	2.127606335
650	1612	OPHSV0400000471	ADAM8	2.12726587
979	2747	H200000333	TRIM26	2.126041844
434	894	H300003660	-	2.125633911
1045	2424	OPHSV0400003286	IER5L	2.125009199
780	1597	H200000037	EPHX2	2.124229664
632	1528	OPHSV0400011021	EFNA5	2.122081218
1209	2975	H300013753	KIAA1984	2.121244678
230	443	H300000630	-	2.118947095
382	715	H200007054	CPSF1	2.117970415
676	1185	OPHSV0400008083	-	2.11787095
343	673	OPHSV0400008586	-	2.115891128
407	757	OPHSV0400005936	NP_001001685.1	2.11402364
524	929	H300008356	NP_775920.1	2.112716257
486	1025	H300013165	SCAND2	2.111790605
3354	7415	H300005800	ABCB5	2.110848581
920	1977	H300006729	HS3ST6	2.110491134
497	1144	H200014083	PSMD7	2.108698368
976	2469	H200004603	TAF13	2.10817147
205	380	OPHSV0400003896	ROR1	2.105492329
482	1128	OPHSV0400012726	-	2.105475832

489	913	OPHSV0400007905	-	2.105275532
1149	2791	H300004096	-	2.10429877
1160	2481	H300019511	TRIM54	2.102885859
3043	7032	H200012945	CAPZA3	2.102700837
1816	3803	H200013363	ZNF544	2.101006599
530	1099	ALIEN2_80	-	2.098776671
315	637	H200011261	NP_001073974.1	2.092635145
196	353	H200011893	CLPX	2.091883901
217	362	OPHSV0400002238	NP_795361.1	2.087131042
305	567	OPHSV0400001505	C21orf87	2.085993673
282	484	H200016932	DNTT	2.08558953
366	726	H300012740	MYO9B	2.085459704
487	936	OPHSV0400012261	-	2.085185121
363	816	H200000438	GGTLA1	2.084665036
575	1078	OPHSV0400011760	-	2.083874363
484	908	H300011227	FMO1	2.083844679
203	405	OPHSV0400012256	-	2.083122053
191	326	H200001128	MUC13	2.083054052
1296	2883	H200018201	SCAMP4	2.082845494
806	1793	H300000382	NKX2-3	2.082410415
2006	5525	OPHSV0400013026	-	2.081415975
10592	27000	OPHSV0400004453	-	2.079086708
196	307	OPHSV0400002358	KCNH7	2.077479895
344	579	OPHSV0400002679	H1FX	2.075990968
193	299	OPHSV0400013247	-	2.075041814
1602	4027	OPHSV0400004479	-	2.074007086
556	1166	OPHSV0400000402	C2orf34	2.073154023
392	810	OPHSV0400001395	MAML3	2.072620946
459	1021	H200014208	VNN3	2.070931692
10321	23060	H200008857	ZNF251	2.070901701
483	910	OPHSV0400004317	Q9H6X1_HUMAN	2.069559735
347	646	H300002930	-	2.069159036
330	617	H200013067	ZNF643	2.067387799
712	1524	OPHSV0400008081	-	2.065516878
1493	3822	H300008935	GRINL1A	2.065174726
756	1849	OPHSV0400011423	-	2.065013415
1623	4006	OPHSV0400010204	-	2.064754748
427	934	H300022642	MDM2	2.064292847
1218	2712	H300008261	LIN54	2.063834305
219	365	H300009255	SCAND1	2.063443217
255	442	H300018403	C1orf124	2.063115323
307	534	OPHSV0400006118	IFITM1	2.062430525
959	1780	H200010941	CCDC65	2.061639936
505	977	H300007495	-	2.060087805
201	286	OPHSV0400000344	MAP3K1	2.059046897
6728	14384	H300011412	MEPE	2.058319757
231	350	H300014178	KLHL3	2.055471936
318	630	OPHSV0400002530	IZUMO1	2.054538995
503	1044	H300019748	SIRPB1	2.054285999
206	379	H200016625	MMRN1	2.054067531
264	478	H200003637	RNF26	2.053164897
206	377	H300019216	SPAG1	2.052992521
373	678	OPHSV0400010660	-	2.051763744
431	995	H300019099	STRAD_HUMAN	2.0511857
553	1236	OPHSV0400009590	-	2.051041893
191	269	H300020974	CCNE2	2.048844971
1345	3061	H200013801	ZBED3	2.048675395
211	326	OPHSV0400003157	-	2.048305132
350	631	H300014156	-	2.048019357
727	1908	H300005566	DNAH2	2.047627323
309	614	H200012407	CASP8AP2	2.045173153
969	2312	H300001680	LOC731890	2.04459417
234	368	H300012282	SOHLH2	2.0442893
703	1673	H300014606	-	2.042843966

231	419	H300020976	CBWD1	2.041410596
537	1201	H300019166	MAPK8	2.039367949
358	659	OPHSV0400003499	-	2.038879549
3194	7329	H300005628	HIST1H3A	2.03771196
385	709	OPHSV0400001358	-	2.03727284
191	319	H200013149	METTL8	2.036628101
3114	6544	H300005511	LY6D	2.03594813
209	347	OPHSV0400006512	MID1	2.035372039
242	391	OPHSV0400008057	-	2.034460415
302	599	H200005458	TFAP2C	2.033993967
905	1847	H200021020	APTX	2.033935684
428	824	OPHSV0400007441	-	2.033564394
1126	2454	OPHSV0400008459	-	2.030763328
926	2253	H200013986	MTHFD2	2.030746345
1493	3097	H200013446	HEY2	2.029490915
196	307	OPHSV0400008868	-	2.028980428
219	361	OPHSV0400010505	-	2.028814531
2802	6189	H300006881	-	2.028477343
499	1092	OPHSV0400006705	Q8N1L4_HUMAN	2.027322998
521	1234	OPHSV04TC000005	-	2.026547191
281	524	H200017636	FGF21	2.023542615
1554	3170	H300018131	-	2.022574
607	1239	OPHSV0400005826	CSAG3B	2.022517544
885	1677	OPHSV0400007079	Q4VXG5_HUMAN	2.022244462
865	1795	H200015474	POU6F2	2.02037546
819	1977	H200014045	ZNF174	2.019355839
908	1839	OPHSV0400003252	DCHS2	2.016750595
202	349	H300011218	-	2.015865985
314	566	H300010870	SLC7A9	2.015688942
3907	6637	OPHSV0400004926	SRPK2	2.015166734
199	351	OPHSV0400006034	-	2.014814769
346	575	H300003865	ZBTB26	2.014578857
993	1993	H200000361	ANXA3	2.01417375
669	1387	H300020381	FAM83A	2.013828509
1265	3032	OPHSV0400003623	-	2.013152903
502	1049	OPHSV0400008701	-	2.012578616
488	944	H300017869	DEGS1	2.011911688
2229	5158	H300002623	OR5BF1	2.011646362
223	336	H200012161	TNNC1	2.008653241
267	446	H200012251	TSP50_HUMAN	2.00660696
219	381	H300017862	FNTB	2.006568194
196	304	H200008341	PSMD14	2.006463994
522	942	H200006784	REPS2	2.00631703
707	1301	H200004533	BATF	2.005919001
3048	7010	OPHSV0400006561	NP_612412.2	2.003307324

Table S2. Down-regulated genes (Zscore <-2)

Cy3	Cy5	Id	Symbol	Zscore
1134	282	H200011432	C10orf39	-3.830372879
407	157	OPHSV0400008678	-	-3.663678364
471	161	OPHSV0400006957	-	-3.535820283
642.3	190.6	H200000680	LDHA	-3.465952508
353	151	H300008109	C1orf150	-3.416334868
257	150	H300012320	PPM1A	-3.364393542
3928	1136	OPHSV0400009798	-	-3.271367217
303	152	OPHSV0400006222	PTTG1	-3.265544895
1904	639	H300017230	MKL2	-3.264221865
2876	864	H300001051	Q8N7N2_HUMAN	-3.192615764
1468	411	H200007673	ATP8B1	-3.154778726
22359	7063	OPHSV0400011094	-	-3.153740546
593	206	H200012662	-	-3.150671123
361	158	OPHSV0400011946	-	-3.123963793
305	152	H300019384	PHGDHL1	-3.111294741

1100	361	H200007022	AXL	-3.101656775
321	156	OPHSV0400011960	-	-3.10143616
280	145	H300022036	C1orf121	-3.088973116
30037	10751	H300007788	Q9UI23_HUMAN	-3.08834304
515	233	H300007745	OR2G3	-3.081825073
1892	551	OPHSV0400009812	-	-3.043857802
307	153	H300017264	RIMBP2	-3.031677916
1984	653	OPHSV0400009796	-	-2.965382672
433	178	OPHSV0400008449	-	-2.95360271
319	151	OPHSV0400012421	-	-2.938145816
344	170	OPHSV0400005179	ZNF100	-2.920291886
3670	1157	OPHSV0400008877	-	-2.8906738
288	153	H300019627	MYEF2	-2.882750653
430	195	H300016858	PEX5L	-2.878661416
21085	7918	H300021296	LBH	-2.871122684
1963	606	OPHSV0400009799	-	-2.865551603
3638	1097	OPHSV0400009797	-	-2.840055637
2504	826	OPHSV0400009811	-	-2.826023944
545	244	OPHSV0400005030	-	-2.777320882
4628	1520	OPHSV0400008672	TOR1AIP2	-2.756802546
516	231	H300008880	OR6T1	-2.754097453
252	146	H300004288	LOC284064	-2.749149213
350	157	H200019704	TBAK_HUMAN	-2.741888025
436	190	OPHSV0400005222	Q6ZS35_HUMAN	-2.739726682
1673	618	OPHSV0400004701	Q6ZND9_HUMAN	-2.739332875
24166	10332	H300007181	USP33	-2.723151697
17645	6666	H200011192	WDR40A	-2.720745844
16115	6734	H300017621	HTR4	-2.719334324
1317	487	OPHSV0400004418	NP_079198.2	-2.717218563
357	176	OPHSV0400012903	-	-2.707070187
274	154	OPHSV0400006415	SMOC1	-2.705049121
300	172	OPHSV0400011956	-	-2.692475909
12005	4539	H200006031	PKIA	-2.685052958
310	159	H300005122	CRYM	-2.675654485
1407	484	OPHSV0400004299	Q68DE7_HUMAN	-2.67391487
1615	544	OPHSV0400009805	-	-2.673133614
1193	521	OPHSV0400009803	-	-2.665493178
508	233	H200011732	NMT1	-2.662586018
39267	12958	H300021492	AGPAT7	-2.661885325
10025	3704	H300004605	NP_057574.2	-2.658480595
403	211	H200019043	-	-2.64396586
1691	565	OPHSV0400008410	RPS14	-2.6397266
2549	949	OPHSV0400009793	-	-2.627947686
1360	543	OPHSV0400004947	Q6PID2_HUMAN	-2.626182941
1335	519	OPHSV0400005331	-	-2.620895676
913	373	H300019778	PFKP	-2.61755737
365	190	H300003191	COX6C	-2.606934787
718	333	OPHSV0400005383	FGFR2	-2.605686778
684	277	H300013194	-	-2.603493568
245	152	OPHSV0400004681	-	-2.597050724
675	293	H300001027	Q15401_HUMAN	-2.59435441
17399	7106	H300006124	OR10G9	-2.592093449
1928	743	OPHSV0400004716	LIN1_HUMAN	-2.585333536
1187	427	OPHSV0400009804	-	-2.582429719
264	149	H200005797	PTDSR	-2.571770904
39081	15446	H300006321	Q8N9C2_HUMAN	-2.567153773
59248	32346	H200018878	PCM1	-2.56561551
244	148	H200007769	OGDH	-2.560685231
680	287	H300002897	CASP9	-2.556624897
274	159	OPHSV0400012189	-	-2.555444251
17380	6721	H300008872	NP_001073961.1	-2.542962832
59867	30312	OPHSV0400007287	-	-2.542535061
10371	4403	OPHSV0400002433	NR4A1	-2.538550488
4852	1706	OPHSV0400005420	ODF2L	-2.535382855

300	169	H300008011	Q7Z2M6_HUMAN	-2.534684392
264	147	OPHSV0400000116	SLC7A14	-2.53293301
1082	481	OPHSV0400004939	-	-2.530789719
445	216	H300022627	BBS1	-2.529891171
286	151	H200012086	MTHFS	-2.523762263
2820	1237	H200005590	SHANK1	-2.522300553
675	321	OPHSV0400000239	ATPBD4	-2.518724341
271	156	H300009634	OR5B2	-2.511724612
391	201	OPHSV0400000504	NP_872374.3	-2.508347051
21628	9096	H300010656	DOT1L	-2.502447427
242	147	OPHSV0400009574	-	-2.498090992
4520	1874	H300004889	LIN1_HUMAN	-2.489253579
32919	14325	H300004373	THOC3	-2.487165804
343	177	H300013045	ADAMTSL3	-2.484502752
244	158	H300007373	THRAP3	-2.481855964
4669	1847	OPHSV0400005225	ODF2L	-2.476611049
8253	2930	OPHSV0400005295	LIN1_HUMAN	-2.475956793
903	408	H200015519	LCE2B	-2.473556923
1224	465	OPHSV0400004834	-	-2.473230464
4546	1859	OPHSV0400004314	-	-2.464181598
6160	2456	OPHSV0400004898	POT14_HUMAN	-2.452003701
879	330	H200015373	MASP1	-2.447216024
239	145	OPHSV0400002176	-	-2.442800144
795	352	H300022210	NXF2	-2.429479394
844	400	H300006975	O00365_HUMAN	-2.419999957
51093	23193	H300007376	CHMP5	-2.414593101
5287	1910	OPHSV0400004483	Q6PID2_HUMAN	-2.407681421
745	363	H300002616	NPY1R	-2.407424333
381	188	OPHSV0400004271	RPL7	-2.403673695
296	175	OPHSV0400008009	-	-2.400194357
653	318	H300000912	-	-2.399982543
12722	4371	OPHSV0400013194	ACOT11	-2.397742623
247	161	H300019884	HIBCH	-2.395653466
320	181	OPHSV0400007917	-	-2.390611875
413	209	H200014659	CCNB2	-2.389846955
3062	1349	H200002363	SEPW1	-2.388868112
1180	560	H300017054	-	-2.388301164
28658	12006	H200014281	CAMK1	-2.388092937
375	201	H300010425	IL22RA2	-2.387551041
14178	5252	H300003050	OR1L1	-2.385853244
42472	16172	H300014197	FXYD2	-2.385823351
355	189	H300007961	FTMT	-2.384523007
19414	7356	H300009440	PDIA6	-2.382125597
690	368	OPHSV0400010820	-	-2.380260414
3169	1271	OPHSV0400006120	-	-2.379372013
3173	1207	OPHSV0400005578	-	-2.376325811
15887	7079	H200011780	SLC30A3	-2.374065901
380	197	H200001908	KLHDC8A	-2.367753861
2315	1061	H300008522	-	-2.365832991
2022	833	H300003717	NNT	-2.365122593
16328	7492	OPHSV0400005152	-	-2.363149
4032	1619	OPHSV0400005094	Q6PID2_HUMAN	-2.360960039
2884	1174	H200014678	O94914_HUMAN	-2.359488097
3297	950	OPHSV0400004878	-	-2.359161934
2515	1173	OPHSV0400002392	NP_001035155.1	-2.35797867
336	181	H300019144	TBXA2R	-2.35762587
252	151	H200010719	SLC2A4	-2.354093269
10337	4231	H200000503	CCNF	-2.352658318
3891	1443	H200010836	LRFN1	-2.349509746
260	151	OPHSV0400005933	Q6ZSQ3_HUMAN	-2.34694506
828	400	H200009334	C8orf41	-2.343846416
6162	2829	OPHSV0400013174	-	-2.339902258
625	297	H300003505	NP_689911.1	-2.336797669
32803	12846	H300007179	-	-2.335180154

1098	505	H300004486	KIAA1161	-2.329219895
17485	6615	OPHSV0400009813	-	-2.328918233
350	193	OPHSV0400002929	hCG_23177	-2.325328563
613	334	OPHSV0400002033	CTAGE1	-2.324578535
10421	4058	H300022310	NP_957704.1	-2.323239964
355	196	OPHSV0400007775	-	-2.322716099
12197	4550	H200012193	ST13	-2.322161703
268	146	OPHSV0400005435	Q15401_HUMAN	-2.31982983
464	226	OPHSV0400006580	ZNF480	-2.315927453
5897	2005	OPHSV0400009810	-	-2.313521911
260	161	OPHSV0400008443	-	-2.312439715
235	150	H300007254	CPXM2	-2.308816078
454	237	OPHSV0400004255	-	-2.307045695
11907	4423	OPHSV0400005558	GAS2L1	-2.30603884
6391	2591	H300017851	-	-2.304772098
37670	16175	H200002589	FBXO3	-2.30462959
2840	1119	OPHSV0400009832	-	-2.299393982
1553	641	OPHSV0400008444	-	-2.299147948
496	250	OPHSV0400012382	-	-2.29689555
235	150	OPHSV0400013035	FAM114A1	-2.295611098
57249	27454	H300008646	O15421_HUMAN	-2.295145131
4671	1932	H300003475	-	-2.291960995
740	335	H200010412	NCALD	-2.290141015
3550	1417	H300008053	Q8N8A5_HUMAN	-2.288558672
308	170	H300004351	ZNF546	-2.288233244
936	398	OPHSV0400004973	-	-2.283560678
308	171	OPHSV0400001798	C21orf57	-2.28293178
380	212	OPHSV0400006421	-	-2.277850369
2213	911	OPHSV0400004581	Q6ZNC3_HUMAN	-2.276828069
247	152	OPHSV0400003969	ACOT11	-2.276289024
6717	3180	H300019491	SLC37A4	-2.275418854
1035	478	H300016009	MED19	-2.275092627
15069	5815	H300011203	-	-2.274703832
454	218	H300021249	C6orf151	-2.274309894
4369	1639	OPHSV0400009826	REV3L	-2.273520827
236	147	OPHSV0400010030	Q4G0H1_HUMAN	-2.27224463
257	160	OPHSV0400011505	-	-2.26732956
30629	12839	H300013003	SLC25A1	-2.264255035
380	204	OPHSV0400005010	-	-2.264152279
293	170	OPHSV0400005538	-	-2.261983731
13808	5740	H300013779	SRPK1	-2.26170652
439	234	H300018673	METTL4	-2.259458965
2143	990	H300000825	Q6ZUR4_HUMAN	-2.258620898
4346	1723	H300004553	OR6M1	-2.256917417
2217	895	OPHSV0400004550	-	-2.254098233
862	420	H300005405	PPIA	-2.253999079
294	173	OPHSV0400006085	MUC2	-2.252810529
12270	5350	H300012246	NP_060028.2	-2.24992702
2054	778	H300011908	-	-2.248739335
244	150	H200006391	FDPS	-2.247896383
522	271	H200008351	TMCC1	-2.246484095
361	200	OPHSV0400002066	-	-2.245884367
6394	2439	H300007222	-	-2.245718717
4488	1648	OPHSV0400004784	ODF2L	-2.244289231
2147	1021	H300016527	-	-2.244074279
237	144	OPHSV0400001095	NTRK3	-2.243545282
329	193	H300006511	ARL3	-2.240561921
346	200	OPHSV0400005245	FGA	-2.238140809
2128	838	H300011817	Q8WZ27_HUMAN	-2.236716397
3050	1173	H300004051	KRTAP9-9	-2.235689603
470	242	H300017811	-	-2.233440177
637	342	H200003217	BCAS2	-2.221920355
728	355	OPHSV0400009546	-	-2.220960106
2402	971	OPHSV0400000173	STK35	-2.220776714

277	164	OPHSV0400010277	OR6C68	-2.220535524
380	208	H200009220	PTGES2	-2.219252895
251	151	OPHSV0400011891	-	-2.218840868
1403	688	OPHSV0400004928	Q9UN78_HUMAN	-2.218053882
299	172	H300009851	-	-2.212374956
3051	1267	OPHSV0400009819	-	-2.209866565
2390	945	H300005088	OR6N2	-2.209365702
7037	2615	H300000092	LIN1_HUMAN	-2.206817527
6895	2882	H300012133	MS4A3	-2.205447063
316	175	H200010401	TBXA2R	-2.204691437
307	192	H300002989	DSG4	-2.203333498
12610	4816	H200006356	DNASE1L1	-2.202138937
5486	2253	H300004377	Q4G127_HUMAN	-2.198302904
1832	766	OPHSV0400004513	LIN1_HUMAN	-2.197634478
249	161	OPHSV0400007173	-	-2.195984865
676	309	OPHSV0400004979	-	-2.194260144
21801	9302	H300005428	SEC11B	-2.194196597
373	213	OPHSV0400005083	-	-2.192097861
674	337	H300017405	O14931-2	-2.190290154
22523	9302	OPHSV0400005582	-	-2.18980444
297	173	OPHSV0400012829	AGPAT4	-2.187618872
3731	1710	H300003711	OR7G1	-2.187507547
540	272	H200016335	TANC1	-2.180546368
1307	464	OPHSV0400004946	-	-2.179776606
2719	1300	OPHSV0400011940	-	-2.177135547
7907	2952	H200013976	SETD1B	-2.176630558
47990	26838	H300022214	WHSC1L1	-2.174341749
3482	1558	H200015122	PFKFB2	-2.174162147
5552	3204	OPHSV0400005147	-	-2.173137606
8868	3371	H200018241	SLC19A3	-2.172726577
1254	547	H300010114	SFT2D1	-2.171390909
3164	1392	H300010201	Q9P155_HUMAN	-2.170578842
42832	24196	OPHSV0400005394	RBM34	-2.170323396
44462	16840	H300005205	Q8NI68_HUMAN	-2.170316123
455	255	OPHSV0400012303	-	-2.170240166
2278	1014	H300017080	CNOT3	-2.169301873
3321	1400	H200007596	ZNF446	-2.168435238
3377	1538	OPHSV0400009807	-	-2.168328149
2894	1412	H300003806	Q8NBL2_HUMAN	-2.166935105
417	210	H200005503	C13orf33	-2.161509535
339	193	H300018224	RBM35A	-2.160168391
2015	793	H300002697	IFNB1	-2.158622993
899	396	OPHSV0400012111	BMPR2	-2.157879897
1090	611	OPHSV0400002321	PDE1A	-2.155268159
3696	1674	H300021329	ZNF672	-2.153768628
29744	11990	H300006510	Q00849_HUMAN	-2.153317833
348	191	H200003148	NP_060541.3	-2.152451374
242	152	H300018164	-	-2.152417251
56021	28032	H300019629	STOML1	-2.151902681
1031	482	OPHSV0400000922	HSPB7	-2.150631572
383	240	H300012891	CHEK1	-2.150249601
639	364	H300007298	-	-2.150247955
10618	4008	H300008167	NEDD8	-2.150046503
22768	11367	H200011551	UHRF1	-2.148347433
6338	3314	H300008274	Q8N2D2_HUMAN	-2.147527972
1734	648	OPHSV0400005716	-	-2.146781698
220	148	H300012834	KCTD9	-2.145544763
2661	1211	OPHSV0400003121	PAX4	-2.142663598
3085	1010	OPHSV0400009806	-	-2.142317093
40278	21024	H200004241	ARCN1	-2.140919527
272	168	H300020404	PAPSS2	-2.14027781
6615	2760	OPHSV0400001643	LHFP	-2.138318451
9331	4313	H300007867	ZNF230	-2.137158449
14600	5789	H300007588	DEFB4	-2.136316907

266	164	OPHSV0400001741	Q9P159_HUMAN	-2.135519681
544	321	H200013452	PIP5K2C	-2.131120299
993	485	H300020950	NP_056129.1	-2.13065578
1490	675	H200007840	ASMT	-2.130431125
1217	556	OPHSV0400004686	-	-2.130249835
3156	1371	OPHSV0400005569	-	-2.127693858
825	429	OPHSV0400011088	-	-2.125092707
260	159	OPHSV0400000662	HPRT1	-2.124570047
41314	19915	H300017185	-	-2.124251827
2407	978	H300009729	-	-2.122536756
230	145	OPHSV0400006324	POGK	-2.122105452
230	145	H200001405	C17orf71	-2.116431428
253	165	OPHSV0400012282	-	-2.112499769
11617	5324	H300013289	-	-2.111235809
606	363	H300006274	TGM2	-2.108167788
1364	688	OPHSV0400004559	-	-2.107748831
4322	1788	H300008824	MT1X	-2.107656458
40628	17320	H300018057	PTGER3	-2.106554358
20804	9641	OPHSV0400011814	-	-2.104580862
1379	657	OPHSV0400005832	OR7E91P	-2.103525937
2203	923	H200014948	KCNK2	-2.101254272
47275	23399	H300022381	ZMYM6	-2.100948237
273	158	OPHSV0400009854	-	-2.100918523
262	149	H300006994	Q49A35_HUMAN	-2.099531231
49333	24769	H300014161	FCHO2	-2.098250628
1287	650	OPHSV0400012714	-	-2.097465134
318	183	OPHSV0400011679	-	-2.096248108
15483	7811	H200017951	STK11	-2.095726867
11166	4410	OPHSV0400004880	Q6ZUR4_HUMAN	-2.091925436
1694	707	OPHSV0400008418	-	-2.091776293
3250	1549	H200006579	CX3CR1	-2.090907318
3495	1570	H300016628	ERCC5	-2.086546694
6648	3023	OPHSV0400004114	KIAA0974	-2.0840034
742	383	H300019554	IPO11	-2.083055395
297	179	OPHSV0400006694	-	-2.082620753
521	276	H200019714	CPM	-2.082603932
2090	1018	OPHSV0400011487	-	-2.081512063
6235.6	2640.6	H200005935	HSPCB	-2.080977634
347	202	OPHSV0400005108	TMCC2	-2.080194963
695	349	OPHSV0400001128	-	-2.079683291
5539	2250	OPHSV0400005844	MT-CO2	-2.076093552
14114	5966	H200015769	-	-2.07403665
12314	5514	H200005597	ZC3H5	-2.073232804
2533	1119	OPHSV0400005256	-	-2.070147048
5669	2574	H200015571	HAO2	-2.069864763
10657	5113	H300022291	ARMC8	-2.066373372
1070	524	OPHSV0400008873	-	-2.066347596
1671	792	H300004556	NP_009174.1	-2.064782907
1663	757	H200016963	C20orf67	-2.064766661
1267	659	H300016954	P25A_HUMAN	-2.063627633
11667	5156	H300008537	ALOXE3	-2.061450312
22445	8414	H300007223	OR2AJ1	-2.060332046
232	147	H200007095	RBM15B	-2.057992406
2076	1040	OPHSV0400001813	Q4G129_HUMAN	-2.055175046
4351	1896	H300004892	Q6ZNC3_HUMAN	-2.053402012
11976	5475	OPHSV0400005291	JOSD1	-2.052095587
1887	862	H300018776	CHCHD2	-2.051574533
245	153	OPHSV0400005344	ANKRD12	-2.050948131
2339	1003	OPHSV04TC000016	CANX	-2.050209916
14300	6852	H300010316	TMEM161B	-2.049547241
1721	905	OPHSV0400007296	-	-2.04739133
862	457	H300019258	OSBPL2	-2.045904075
246	162	H300006690	SLC36A1	-2.045619983
4358	2361	H300011874	CS029_HUMAN	-2.044433986

4429	1785	OPHSV0400005561	-	-2.044157665
9995	3956	H300000517	ARSK	-2.043862149
1208	626	H300018305	AKT2	-2.043036936
701	395	OPHSV0400011385	-	-2.042857079
362	217	OPHSV0400007494	NP_001017370.1	-2.042023161
341	196	ALIEN10_60	-	-2.040821892
11562	5535	H300010282	ANGEL2	-2.039462505
758	403	H300020041	EIF4H	-2.037637131
5772	3006	H300003497	-	-2.036373592
26750	10794	H300007221	-	-2.036269782
1092	573	OPHSV0400011350	-	-2.035633075
4320	2168	H200005990	MARCKSL1	-2.035071282
3020	1032	OPHSV0400005004	LIN1_HUMAN	-2.03401673
303	190	OPHSV0400006895	-	-2.033314034
941	476	H300009690	OR51A2	-2.033295573
1759	938	H200011369	GFRA1	-2.031668738
372	209	H300006287	TUBAL3	-2.031503633
205	144	H300008855	Q6UXG0_HUMAN	-2.030585994
1583	830	OPHSV0400003117	ANKRD30A	-2.028288738
413	232	OPHSV0400004593	-	-2.025882004
581	321	H300005064	-	-2.025769581
5026	2152	H300002453	OR5U1_HUMAN	-2.025068269
8643	4017	H300021735	STAG3	-2.024131297
1657	741	OPHSV0400011567	-	-2.022884919
1241	680	OPHSV0400012788	-	-2.021212179
3259	1638	OPHSV0400012476	-	-2.017302919
777	391	H200017608	FABP2	-2.016419664
2803	1176	H300011229	NP_001073999.1	-2.016136026
3878	1565	H300011483	SAKS1_HUMAN	-2.015818624
1118	563	H200000500	APCS	-2.014083613
20371	8657	H300008829	Q8N8B3_HUMAN	-2.013184646
5861	2364	H200014206	CRABP2	-2.012977205
274	166	H200014555	CPLX2	-2.012966658
546	308	OPHSV0400006145	DDX6	-2.012941251
641	335	H200017648	EPS15L2	-2.012726089
1050	568	H200018907	TRIM22	-2.01180511
263	162	H200018030	CCRL2	-2.011540996
6868	2995	H200007762	TAS2R1	-2.01128449
1760	891	H300013640	NRG1	-2.010365277
1289	602	H200007945	DIRC1	-2.009691822
515	282	H200008648	ARL4D	-2.009507626
805	451	H300006447	MT1G	-2.009391603
1252	599	OPHSV0400005926	NP_001001663.1	-2.009214035
425	230	OPHSV0400012730	-	-2.008325166
10889	4899	OPHSV0400012587	-	-2.007065209
554	307	H200010499	PSORS1C1	-2.007031403
254	165	OPHSV0400006387	NP_065895.1	-2.005363308
18832	8077	H300002888	MDM4	-2.004629277
1114	584	H200015781	LYG6C_HUMAN	-2.004418218
2607	1321	OPHSV0400012543	-	-2.004332598
6215	2725	OPHSV0400007302	-	-2.00357724
3617	1628	H200011790	-	-2.003250426
295	190	H200013086	WDR31	-2.002947677
2028	1007	OPHSV0400003030	PNPLA2	-2.002460158
302	197	H200003679	NP_078880.1	-2.002386669
409	228	H200001821	SLC35F2	-2.002298334
2395	1164	H300017573	NP_001073877.1	-2.001650445
5945	2589	H200004635	ATP8B2	-2.000532892
614	325	H300014300	-	-2.000100923
